# Bayesian predictors of very poor health related quality of life and mortality in patients with COPD

**DOI:** 10.1186/1472-6947-13-34

**Published:** 2013-03-07

**Authors:** Olli-Pekka Ryynänen, Erkki J Soini, Ari Lindqvist, Maritta Kilpeläinen, Tarja Laitinen

**Affiliations:** 1 Department of Public Health and Clinical Nutrition, University of Eastern Finland, Kuopio, Finland; 2 General Practice Unit, Kuopio University Hospital, Primary Health Care, Kuopio, Finland; 3 ESiOR Oy, Kuopio, Finland; 4 Department of Social and Health Management, University of Eastern Finland, Kuopio, Finland; 5 Phoru, School of Pharmacy, University of Eastern Finland, Kuopio, Finland; 6 Department of Medicine, Division of Pulmonary Medicine, Helsinki University Central Hospital.School of Pharmacy, University of Eastern Finland, Kuopio, Finland; 7 Department of Pulmonary Medicine and Clinical Allergology, Turku University Central Hospital, Turku, Finland

**Keywords:** Chronic obstructive pulmonary disease, Bayesian prediction, Bayesian methods, Prognosis, Mortality, Quality of life, Survival analysis

## Abstract

**Background:**

Chronic obstructive pulmonary disease (COPD) is associated with increased mortality and poor health-related quality of life (HRQoL) compared with the general population. The objective of this study was to identify clinical characteristics which predict mortality and very poor HRQoL among the COPD population and to develop a Bayesian prediction model.

**Methods:**

The data consisted of 738 patients with COPD who had visited the Pulmonary Clinic of the Helsinki and Turku University Hospitals during 1995–2006. The data set contained 49 potential predictor variables and two outcome variables: survival (dead/alive) and HRQoL measured with a 15D instrument (very poor HRQoL < 0.70 vs. typical HRQoL ≥ 0.70).

In the first phase of model validation we randomly divided the material into a training set (n = 538), and a test set (n = 200). This procedure was repeated ten times in random fashion to obtain independently created training sets and corresponding test sets. Modeling was performed by using the training set, and each model was tested by using the corresponding test set, repeated in each training set. In the second phase the final model was created by using the total material and eighteen most predictive variables. The performance of six logistic regressions approaches were shown for comparison purposes.

**Results:**

In the final model, the following variables were associated with mortality or very poor HRQoL: age at onset, cerebrovascular disease, diabetes, alcohol abuse, cancer, psychiatric disease, body mass index, Forced Expiratory Volume (FEV_1_) % of predicted, atrial fibrillation, and prolonged QT time in ECG. The prediction accuracy of the model was 77%, sensitivity 0.30, specificity 0.95, positive predictive value 0.68, negative predictive value 0.78, and area under the ROC curve 0.69. While the sensitivity of the model reminded limited, good specificity, moderate accuracy, comparable or better performance in classification and better performance in variable selection and data usage in comparison to the logistic regression approaches, and positive and negative predictive values indicate that the model has potential in predicting mortality and very poor HRQoL in COPD patients.

**Conclusion:**

We developed a Bayesian prediction model which is potentially useful in predicting mortality and very poor HRQoL in patients with COPD.

## Background

Due to its ever increasing prevalence, chronic obstructive pulmonary disease (COPD) has become a global health priority and a significant burden on the health care system. With worldwide prevalence of 8–20%, depending on which definition is used [1], it has been estimated that COPD will be the third leading cause of death by 2020 [2]. The principal goals of management of COPD are to reduce symptoms, prevent exacerbations, and maintain the patient’s physical and emotional capabilities, and thereby improve the patient’s health-related quality of life (HRQoL).

Assessment of HRQoL could be an important tool in the management and monitoring of COPD due to chronic, non-curable, and usually progressive nature of the disease. So far, there is no single ‘state-of-the-art’ approach. We have previously validated two questionnaires on COPD: the disease-specific AQ20 focusing solely on respiratory health and 15D which has a generic approach [3,4]. We found that the compliance rates and measurement properties of both questionnaires were to a large extent comparable and thus, either of them could easily be used in clinical practice to monitor potential progression of the disease.

In clinical trials, the underlying diseases are often excluded, the severity staging of COPD is often limited, and female patients under represented. In real world, however, the severity of COPD varies considerably, several phenotypes are recognized [5], and COPD is often associated with a wide variety of co-morbidities which might be of extreme importance for the patient’s well-being and prognosis [6]. There is growing evidence that the disease phenotype differs between genders [7-11] and women often report lower HRQoL than men [12].

Several recently published articles have concentrated on predictive factors of COPD mortality [13-16], or hospitalization [17], or both [18]. Tsimogianni et al. (2009) [19] studied predictive factors for length of stay in a hospital and 3-year mortality. The following factors have been found to be associated with mortality risk: St George’s respiratory questionnaire total score, the mental and physical components of the SF-36 HRQoL scale [13], depressive symptoms [14], smoking [15], co-morbidity [15], age and inspiratory capacity [16] and PaO_2_[15,16], low forced expiratory volume in 1 s (FEV_1_) [16], and increased body mass index [16,19]. Older age, FEV_1%_ of predicted, emergency room visits for COPD during the previous year, cardiovascular co-morbidity, and prednisone use at the baseline were associated with a greater risk of hospitalization [17]. Scoring on the Medical Research Council chronic dyspnea scale has been proved to be predictive of length of stay in a hospital [19]. These predictions have mostly been made using regression methods.

Recently, Himes et al. (2009) [20] used Bayesian models to predict the development of COPD in asthma patients, finding age, sex, ethnic background, smoking history, and eight co-morbidities to predict the development of COPD. The prediction accuracy was excellent: the area under the ROC curve was 0.83. The strongest predictor of COPD was age.

The aim of this study was to develop a naïve Bayesian classification (NBC) merger model (NBCMM) by which we could identify the risk factors for mortality and very poor HRQoL in a large real world COPD patient cohort. We included to the model the most essential lung function measurements, patients’ health behavior, and common co-existing chronic diseases in the patient profile.

## Methods

### Subjects

Hospital Discharge Registries were used to identify all patients with COPD who had visited the Pulmonary Clinics of the Helsinki and Turku University Hospitals (some 700 000 inhabitants live in the area) during the years 1995**–**2006. The databases were screened by ICD10 code J44.8 and contained all patients between 18 to 75 years of age. All identified patients were invited to the study without further selection. The recruitment was done through a two-phase mailing campaign, through which altogether 844 patients participated to the study. The overall response rate was 27%. The research visits occurred during the years 2005**–**2007. All the participants gave their informed consent which allowed the research consortium to collect, merge, and analyze their comprehensive medical history from all the healthcare providers who had treated them during the past 5**–**10 years and agreed to continue their follow-up on an annual basis for the next 10 years.

All the hospitals, health care centres, and other outpatient clinics that had treated the patients were contacted to archive a complete, unbroken medical history for each participant. The patients’ social security number was used to combine the data from different sources. Source data with personal identifiers was managed and stored in the Clinical Research Centres administered by the Helsinki and Turku University Central Hospitals. From the medical records we identified the results of flow-volume spirometry including bronchodilatation tests, weight and height, ECGs at rest, and smoking status of the patient, and arterial blood gas analysis results. The most up-to-date results were used in the analysis. The reference values for FEV1 (forced expiratory volume in 1 second) and FVC (forced vital capacity) used in Finnish clinical practice are validated in large Finnish population samples consisted of both genders and a wide range of age group [21]. All the given diagnoses stated in the medical records were carefully evaluated, especially when the time of onset and certainty of the diagnosis was determined.

In the data, category ‘coronary disease’ included the patients who have had a myocardial infarct, acute coronary syndrome, or angina pectoris diagnosed by an internist. ‘Cerebrovascular diseases’ included patients with strokes and transient ischemic attacks diagnosed by a neurologist. ‘Cardiovascular diseases’ consisted of patients having one of the following diseases: coronary, cerebrovascular disease, or peripheral artery occlusive disease. Category ‘diabetes’ includes patients with the type 1 and 2 disease. Chronic alcoholism, alcohol use disorder, and treatment of an alcohol use related disorder were all categorized as ‘alcohol abuse’. A wide range of psychotic disorders and long lasting clinical depression and anxiety with a need for regular medication were categorized as ‘psychiatric disease’. Category ‘cancer’ included all malignant solid tumors and malignant haematological diseases. Annual deaths were determined from the national population registry.

The study approach was approved by the Coordinating Ethics Committee of the Helsinki and Uusimaa Hospital District and permission to conduct this research was granted by the Helsinki and Turku University Hospitals.

### Assessment of HRQoL and clinical measures

HRQoL was assessed using the self-administered generic 15D instrument [3]. All participants filled in during the research visit without any guidance from the research personnel. The 15D instrument can be used primarily as a single index measure ranging from a minimum score of 0 to a score of 1 for perfect health, but also as a profile measure. It describes health status with mobility, vision, hearing, breathing, sleeping, eating, speech, elimination, usual activities, mental function, discomfort and symptoms, depression, distress, vitality, and sexual activity. More generally, in most of the important properties (reliability, validity, discriminatory power, and responsiveness) the 15D instrument compares at least equally with other commonly-used preference-based generic HRQoL instruments such as EQ-5D, SF-6D, and HUI3 [3,22,23].

To categorize the patients as having either lower (very poor) or higher (typical) HRQoL, we used as the cut off –point one standard deviation under the mean of the total score. Thus, patients with the total score ≤ 0.70 (N=175, 27%) in 15D were determined as having very poor HRQoL. In a Finnish study using 15D in consecutive patients in secondary care, 15D index 0.70 was the mean result of patients who reported their general health as "very poor" [24]. Rest of the patients formed the group of typical HRQoL. In mortality model, all cause deaths were analysed as the end point.

### Bayesian classification

PREQ, a web-based NBC, was used in the modeling. PREQ is a Bayesian classifier which is able to use multidimensional priors, e.g. separate priors for the outcome variable in general and for the outcome variable according to each predicting variable. NBCs have equaled or outperformed logistic regressions, especially in small data sets, in terms of prediction accuracy [25,26], variable selection, and multiple performance measures [26]. Modeling of this data was done without informative *a priori* information.

The precision of the model refers to its capability to predict observations in the learning set precisely, and reliability refers to the model’s capability to predict observations in the test set or future data set which are not included in model learning. There is a noted trade-off between precision and reliability [26], and we searched for a model with acceptable performance in both metrics. We assessed these with portioning [27], in which the data set was divided into independent learning and test sets.

In order to build a NBCMM with reasonable precision and reliability, we did 10 cross validation models (portioning). In the first phase, we randomly divided the material into a training set (n_1_ = 538) and a test set (n_2_ = 200). This procedure was repeated nine times to get 10 randomly created training sets and corresponding test sets. Each training and test set contained one outcome variable (HRQoL ≤ 0.70 or dead/living with HRQoL > 0.70) and 49 potential predictive variables, being both numerical and categorical variables. Only baseline variables have been used for prediction.

A Bayesian classification was performed for each training set and the created model was tested by using the corresponding test set (i.e., the test were performed with patients not used in the model building). Thus, we obtained 10 different predictive models for a NBCMM model, each tested both internally using the teach set and externally using the test set.

To select the most predictive variables, only those variables present in at least four training set models (likely variables) were accepted into the final model building. After this procedure, we had a set with one outcome variable and 18 potential predictive variables. These variables were used to create a final NBCMM. The validity of this NBCMMwas assessed using common leave-one-out cross-validation (LOOCV [24]).

We assessed the relationships between class variables and predictors with posterior odds (PO), which equal the product of the prior odds and the likelihood ratio, and also estimated inversed probabilities, giving an idea of the predictor’s strength. The POs were

$$\mathrm{PO}=P_{\mathrm{PC}}/P_{\mathrm{NPC}}$$

In which P_PC_ represents the predicted class and P_NPC_ the non-predicted class. POs are not directly dependent on data size, and they give an idea of the predictor’s strength [24]. The credibility intervals (CrI) for accuracies and POs were estimated using the Jeffreys interval [26,27]. Specificity, sensitivity, positive predictive value, negative predictive value, and area under the ROC curve were also established.

In addition, the classification performance of obtained NBCMM was compared to the classification performance of common logistic regression (logit) models. Six logit models were implemented in Stata v10: Logit1 was based on all variables included in the NBCMM (this was comparable to the NBCMM in terms of included predictors), logit2 was based on variables which had at least 80% of observations present, logit3 was based on variables which had 100% of observations present (this was comparable to the NBCMM in terms of number of observations included), logit4 is based on forward stepwise elimination with p-value 0.10 threshold for the predictors, logit5 is based on backward stepwise elimination with p-value 0.10 threshold for the predictors (this shows how well the greedy hill-descending predictor selection [26] and NBCMM approach performed in comparison to the p-value selection), and logit6 is based on manual one-by-one dropping of the predictor variable with the poorest p-value until all logit predictors have a p-value below 0.10.

## Results

### Patient recruitment and selection

A total of 844 patients participated in the study. The COPD diagnosis was re-evaluated. This evaluation led to the exclusion of 105 patients (explained in detail in previous study) [28]. Thus, a final cohort of 739 eligible patients with smoking-related symptomatic chronic bronchitis was included in the modeling. Based on their retrospective medical records, the clinical and demographic findings of participants are shown in Table 1 on average 5.5 years prior to the evaluation of their HRQoL by using the 15D instrument (Table 1). According to the GOLD criteria, a majority of the patients belonged to stages 2–3, i.e. moderate to severe COPD. So far the cohort has been monitored 1–3 years. After the first follow-up year a total of 49 patients (4.0%) had deceased.

**Table 1:** Comparison of the demographic and clinical characteristics of the COPD patients with very poor or typical HRQoL measured with the generic 15D instrument

**Characteristics**	**Very poor HRQoL N = 152**	**Typical HRQoL N = 587**	**P value**
Male gender, N (%)^1^	93 (61.2)	377 (65.1)	0.4
Age, mean in years (±SD) ^2^	63.6 (7.0)	64.1 (6.8)	0.3
Duration of COPD, mean in years (±SD) ^2^	6.4 (4.3)	5.3 (5.0)	0.002
Age at asthma onset, mean in years (±SD) ^2^	52.8 (7.3)	57.6 (7.3)	0.002
Age at COPD onset, mean in years (±SD) ^2^	57.6 (7.6)	58.4 (7.3)	0.2
Pack years, mean (±SD) ^2^	49 (19)	51 (20)	0.4
DLcCOVA % of predicted, mean (±SD) ^2^	74 (23)	77 (22)	0.3
Baseline spirometry ^2^			
FEV_1_% of predicted	52.5 (21.1)	58.4 (18.0)	<0.0001
FVC% of predicted	68.1 (19.0)	76.2 (17.6)	<0.0001
FEV_1_/FVC of predicted, mean (±SD)	61.6 (15.7)	61.5 (13.4)	0.7
Spirometry after bronchodilatation ^2^			
FEV_1_% of predicted	57.7 (20.3)	61.7 (16.8)	0.04
FVC% of predicted	71.5 (17.9)	79.1 (16.9)	<0.0001
FEV_1_/FVC of predicted, mean (±SD)	64.3 (14.8)	62.9 (13.2)	0.2
Proportion (%) of patients at GOLD ^1^			
former stage 0: FEV_1_≥80%	11.2	9.5	
stage 1: FEV_1_/FVC<70%, FEV_1_≥80%	1.0	3.0	
stage 2: FEV_1_/FVC<70%, 50%≤FEV_1_<80%	24.5	40.4	
stage 3: FEV_1_/FVC<70%, 30%≤FEV_1_<50%	18.4	18.0	
stage 4: FEV_1_/FVC<70%, FEV1<30%	9.2	4.3	
stage undefined: FEV_1_/FVC≥70%, FEV_1_<80%	35.7	24.8	0.02
Proportion (%) of patients with home oxygen therapy	8.6	1.7	<0.0001
In severe COPD (FEV_1_<50% of predicted) ^2^			
Arterial O_2_ pressure, mean (±SD)	9.0 (1.5)	9.7 (14)	0.006
Arterial CO_2_ pressure, mean (±SD)	5.5 (0.9)	5.3 (0.5)	0.4
Proportion (%) of deaths during follow-up^1^	9.9	5.5	0.05
HRQL scores, mean (±SD) ^2^			
Airway-specific (range 20–0)	13.0 (3.6)	6.9 (4.5)	<0.0001
Generic (range 0–1)	0.63 (0.06)	0.83 (0.07)	<0.0001
Proportion (%) of patients ^1^			
Underweight BMI < 20	1.4	3.1	
Normal weight BMI 20–25	33.6	41.2	
Overweight BMI 25–30	53.6	50.1	
Obese BMI > 30	11.4	5.6	0.04
Proportion (%) of patients with co-commitant ^1^			
Coronary disease	27.0	20.2	0.07
Cerebrovascular disease	12.5	6.2	0.009
Cardiovascular disease	37.5	25.6	0.004
Diabetes	25.7	12.4	<0.0001
Peripheral artery occlusive disease	3.9	4.7	0.7
Metabolic syndrome	32.9	19.0	0.001
Alcohol abuse	21.7	13.3	0.01
Psychiatric condition	17.8	8.3	0.001
Hypertension	46.7	39.0	0.09

^1^Chi-square test, ^2^Mann-Whitney U test.

The following demographic and clinical variables were recorded: age, gender, duration of the disease, body mass index (BMI), current smoking status, package years, results of the baseline and post-bronchodilatation spirometry and single breath diffusing capacity corrected by hemoglobin concentration and alveolar volume (DLcCOVA), ECG trace, and common co-existing diseases (Table 2). The predictors of very poor HRQoL (15D total score <0.70) were assessed using the 15D instrument. We also studied the clinical predictors of all cause death during the follow-up period.

**Table 2:** Predicting variables present in ten random sets and in the final model

**Variable**	**Definition of variable**	**Model number**										
		**1**	**2**	**3**	**4**	**5**	**6**	**7**	**8**	**9**	**10**	**Final**
Gender	male/female		✓	✓	✓					✓		
Current smoking	reported smoking yes/no		✓		✓							
Year of onset of COPD	numerical variable			✓						✓	✓	
Age at onset of COPD	numerical variable								✓			
Age at onset of asthma	numerical variable			✓								✓
Duration of COPD	numerical variable		✓		✓							
Hypertension	diagnosed hypertension with medication yes/no			✓					✓		✓	
Cerebrovascular disease	diagnosed cerebrovascular disease yes/no	✓				✓	✓	✓		✓	✓	✓
Peripheral artery occlusive disease	diagnosed peripheral artery occlusive disease yes/no								✓			
Any cardiovascular disease	diagnosed any cardiovascular disease yes/no			✓			✓		✓	✓		
Diabetes	diagnosed diabetes yes/no	✓	✓	✓	✓			✓	✓			✓
Metabolic syndrome	diagnosed metabolic syndrome yes/no	✓		✓			✓				✓	
Hypercholesterolemia	diagnosed hypercholesterolemia with medication yes/no								✓	✓		
Hypothryreosis	diagnosed hypothyreosis with medication yes/no			✓							✓	
Alcoholism	diagnosed alcoholism yes/no	✓	✓	✓	✓	✓		✓	✓	✓	✓	✓
Cancer	diagnosed cancer yes/no	✓	✓	✓	✓				✓	✓	✓	✓
Atrial fibrillation	diagnosed permanent atrial fibrillation yes/no	✓	✓	✓	✓	✓					✓	✓
Psychiatric disease	diagnosed any psychiatric disease yes/no		✓	✓	✓	✓	✓		✓	✓	✓	✓
Body mass index	1. BMI < 20					✓	✓	✓		✓		✓
	2. BMI 20–25											
	3. BMI 25.1 – 30											
	4. BMI > 30											
Body mass index change	numerical variable		✓	✓	✓	✓						
DLcCOVA % of predicted	numerical variable			✓						✓		
RV % of predicted	numerical variable						✓				✓	
FVC baseline % of predicted	numerical variable			✓						✓	✓	
FEV_1_ at baseline % of predicted	numerical variable					✓	✓	✓	✓			✓
QRS axel in ECG	numerical variable	✓								✓		
QTc duration in ECG	numerical variable		✓		✓		✓	✓		✓	✓	✓
QRS duration in ECG	numerical variable					✓						

In the first phase we constructed prediction models by using the randomly selected training sets. These models were separately compared with the corresponding test sets. The diagnostic values of ten random training sets compared with the corresponding test sets are presented in Table 3. The proportion of subjects having very poor HRQoL or who died varied between 25.8% – 30.5% (mean 28.7%). The diagnostic values of these models, derived from the accuracy and predictive values, were at a good level, but the mean sensitivity of the model was only 19.5%.

**Table 3:** Diagnostic values of ten random training sets compared to corresponding test sets

**Training set number**	**Sensitivity**	**Specificity**	**Diagnostic accuracy**	**Positive predictive value**	**Negative predictive value**
1	23.4	95.4	78.5	61.1	80.2
2	15.6	94.8	77.0	46.7	79.5
3	21.7	93.5	77.0	50	80
4	16.7	96.1	77.0	57.1	78.5
5	30.2	87.8	72.5	47.1	77.7
6	12.1	96.5	72.0	58.3	72.9
7	15.4	92.6	72.5	42.1	75.7
8	4.2	97.4	75.0	33.3	76.3
9	41.1	85.4	73.0	52.3	78.9
10	14.4	88.1	67.0	32	72
**mean**	**19.5**	**92.8**	**74.2**	**48**	**77.2**
standard deviation	10.3	4.2	3.4	9.9	2.9

**Figure 1:**
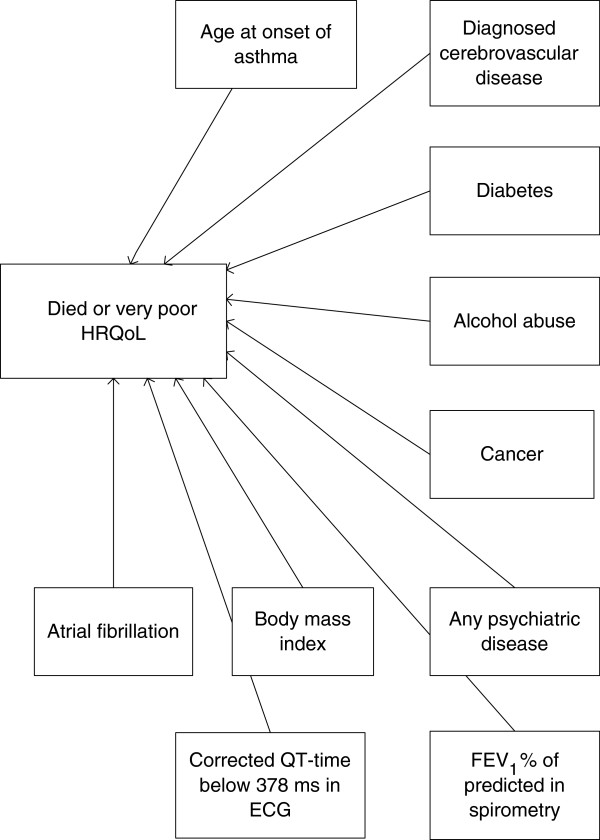
Final NBC model for predicting death or living with very poor health-related quality of life.

In the final model, the following variables were associated with mortality or very poor HRQoL: age at onset of COPD, diagnosed cerebrovascular disease, diabetes, alcohol abuse, cancer, any psychiatric disease, body mass index, FEV_1_% of predicted in spirometry, atrial fibrillation, and corrected QT time below 378 ms in ECG. The final model is presented in graphical form in Figure 1.

Posterior distributions and inversed probabilities of the final model using a NBC according to each predictive variable are presented in Table 4. The results indicate that patient age over 65 years at asthma onset, normal body mass index, and FEV_1_ over 89 are strong factors that protect against death or very poor HRQoL. Other factors in the model are suggestive of a protective effect. The same factors do not work as risk factors, as seen when POs as protective factors are compared with figures of risk factors in Table 4. The absence of comorbidity (diabetes, alcoholism, cancer, psychiatric disease, atrial fibrillation) is a strong protective factor against death or very poor HRQoL. Thus, the model is good in predicting health, but there is no good value for risk factors in predicting disease.

**Table 4:** Main results: posterior distributions and inversed probabilities of the final NBCMM using a NBC

**Factors of very poor QoL/death**	**Predicted class**						**Evidence strength**					
***Protective***	***Alive with not very poor QoL***						***Inversed (If alive with not very poor QoL)***					
	***%***	***95% CrI (%)***		***PO***	***95% CrI***		***%***	***95% CrI (%)***		***PO***	***95% CrI***	
Age at asthma onset over 65.49	80.0	76.9	83.0	4.0	3.3	4.9	16.0	13.4	19.1	0.2	0.2	0.2
No CVD	77.0	73.6	80.1	3.3	2.8	4.0	76.0	72.6	79.2	3.2	2.6	3.8
No diabetes	76.0	72.6	79.2	3.2	2.6	3.8	88.0	85.3	90.3	7.3	5.8	9.3
No alcoholism	75.0	71.5	78.2	3.0	2.5	3.6	87.0	84.3	89.4	6.7	5.4	8.4
No cancer	74.0	70.6	77.3	2.8	2.4	3.4	96.0	94.3	97.3	24.0	16.5	36.0
No psychiatric disease	75.0	71.5	78.2	3.0	2.5	3.6	93.0	90.9	94.8	13.3	10.0	18.2
BMI 0	87.0	84.3	89.4	6.7	5.4	8.4	3.0	1.8	4.4	0.0	0.0	0.0
FEV_1_ over 89.61	81.0	77.8	83.9	4.3	3.5	5.2	5.0	3.5	6.8	0.1	0.0	0.1
No AF	74.0	70.6	77.3	2.8	2.4	3.4	95.0	93.2	96.5	19.0	13.7	27.6
QT below 378.17	71.0	67.4	74.3	2.4	2.1	2.9	4.0	2.7	5.7	0.0	0.0	0.1
***Risk***	***Very poor QoL/death***						***Inversed (If very poor QoL/death)***					
	***%***	***95% CrI (%)***		***PO***	***95% CrI***		***%***	***95% CrI (%)***		***PO***	***95% CrI***	
Age at asthma onset below 45.53	47.0	43.2	50.8	0.9	0.8	1.0	18.0	15.1	21.0	0.2	0.2	0.3
CVD	37.0	33.3	40.7	0.6	0.5	0.7	38.0	34.3	41.8	0.6	0.5	0.7
Diabetes	43.0	39.2	46.8	0.8	0.6	0.9	24.0	20.8	27.4	0.3	0.3	0.4
Alcoholism	40.0	36.3	43.4	0.7	0.6	0.8	23.0	19.9	26.4	0.3	0.2	0.4
Cancer	49.0	45.2	52.8	1.0	0.8	1.1	10.0	7.9	12.5	0.1	0.1	0.1
Psychiatric disease	46.0	42.2	49.9	0.9	0.7	1.0	17.0	14.3	20.0	0.2	0.2	0.3
BMI 3	51.0	47.2	54.8	1.0	0.9	1.2	12.0	9.7	14.7	0.1	0.1	0.2
FEV_1_ below 31.11	50.0	46.2	53.9	1.0	0.9	1.2	12.0	9.7	14.7	0.1	0.1	0.2
AF	47.0	43.2	50.8	0.9	0.8	1.0	11.0	8.7	13.6	0.1	0.1	0.2
QT over 506.59	98.0	96.7	98.9	49.0	29.3	89.9	3.0	1.8	4.5	0.0	0.0	0.0

When the total material was fitted into the model, the following results were achieved: prediction accuracy 77%, sensitivity 0.30, specificity 0.95, positive predictive value 0.68, negative predictive value 0.78, and area under the ROC 0.69. In LOOCV, the default classification score (best guess) was 72.0% (log-score 0.583) and the model’s performance with selected variables was 75.4% (corresponding log-score 0.536).

Table 5 shows the results of logit models. The logit1 with correspondent predictors to NBCMM had marginally higher classification accuracy in comparison to the NBCMM. However, the logit1 used only 56% of the 647 observations in the data set and may have classification accuracy between 44–78% if all observations were modeled. The logit2 had relatively similar classification accuracy in comparison to the NBCMM. It used 85% of the observations in the data set and may have classification accuracy between 66-78% if all observations were modeled. The logit3 with all observations used had lower classification accuracy (72%) in comparison to the NBCMM.

**Table 5:** Logistic regression models with six different approaches

**Very poor HRQoL/death outcome**	**Logit 1**		**Logit 2**		**Logit3**		**Logit 4**		**Logit5**		**Logit6**	
**Parameter**	**OR**	**SE**	**OR**	**SE**	**OR**	**SE**	**OR**	**SE**	**OR**	**SE**	**OR**	**SE**
Age at onset of COPD	0.9772	0.0178	0.9860	0.0148			0.8432*	0.0585			0.9342*	0.0273
Age at onset asthma									0.8469*	0.0560		
Year of birth							0.8579	0.0680	0.8663	0.0647	0.9163*	0.0313
Diagnosed cerebrovascular disease	2.2090	0.9669	2.4865*	0.8858	2.5121**	0.7810						
Diabetes	2.0537*	0.6558	2.2233**	0.6105	2.2204**	0.5260						
Alcohol abuse	2.1063*	0.6814	2.4580**	0.6461	1.9996**	0.4726					2.4388**	0.7556
Cancer	2.5036	1.3626	2.1930**	1.2808	2.5107*	0.8961						
Any psychiatric disease	4.6815***	1.7979	3.3283***	1.0637	2.5081**	0.7054	3.3262	2.4100	4.0301	2.9826	3.8206***	1.4034
Body mass index	1.2048	0.2493	1.4440*	0.2496								
FEV1% of predicted spirometry	0.9761**	0.0071	1.4440***	0.0580							0.9851*	0.0065
Atrial fibrillation	3.1544*	3.1544									2.6895*	1.1314
Corrected QT-time	1.0093*	0.0047										
Tests	Value	p	Value	p	Value	p	Value	p	Value	p	Value	p
N included to the model	365		547		647		75		75		389	
Probability of the model (chi^2^)	52.90	<0.0001	72.00	<0.0001	48.21	<0.0001	10.25	0.0166	10.28	0.0163	36.94	<0.0001
Pseudo R^2^	12.18%		11.69%		6.38%		12.07%		12.11%		7.88%	
Sensitivity	33.01%		24.09%		15.43%		21.05%		21.05%		21.24%	
Specificity	95.80%		95.61%		93.64%		94.64%		94.64%		94.20%	
Positive predictive value	75.56%		64.71%		47.37%		57.14%		57.14%		60.00%	
Negative predictive value	78.44%		79.03%		74.92%		77.94%		77.94%		74.50%	
Log likelihood	−190.73		−271.87		−353.57		−37.32		−37.31		−215.93	
Akaike information criteria	403.46		561.74		719.14		82.65		82.61		445.87	
Bayesian information criteria	446.36		600.48		745.98		91.92		91.88		473.61	
Proportion of observations used	56.41%		84.54%		100.00%		11.59%		11.59%		60.12%	
Correct classification of used N	78.08%		77.70%		72.49%		76.00%		76.00%		73.01%	
Worst possible correct classification among all (N 647)	44.05%		65.69%		72.49%		8.81%		8.81%		43.89%	

NBCMM = naïve Bayesian classification merger model. Logit1 = all variables included in the NBCMM (comparable to the NBCMM in terms of predictors included to the logit model). Logit2 = variables which had at least 80% of observations present. Logit3 = variables which had 100% of observations present (comparable to the NBCMM in terms of number of observations included). Logit4 = forward stepwise elimination with p-value 0.10 threshold. Logit5 = backward stepwise elimination with p-value 0.10 threshold (shows how well the greedy hill-descending predictor selection of naïve Bayes approach performed in comparison to the p-value selection). Logit6 = manual one-by-one dropping of the predictor variable with the poorest p-value until all logit predictors have a p-value below 0.10. *p<0.050. **p<0.010. ***p<0.001.

The logit4 and logit5 (stepwise machine learned models based on p-values) had marginally lower classification accuracy (76%) in comparison to the NBCMM. However, they used only 12% of the observations in the data set, ignored many clinically meaningful predictors and may have classification accuracy between 9–76% if all observations were modeled. The logit 6 (manually selected predictors based on the p-values) had lower classification accuracy (73%) in comparison to the NBCMM. Yet, the logit6 used only 60% of the observations in the data set and may have classification accuracy between 44–73% if all observations were modeled. The poorer performance of p-value based variable selection for the logit model (stepwise or manual selection) in comparison to the greedy hill-descending algorithm of NBC may result from the complex nature of the data set (e.g. cross-censoring present in the variable values).

The sensitivity of the NBCMM was limited in this case. However, good specificity, moderate prediction accuracy, comparable or higher accuracy as well as more efficient variable selection and data usage in comparison to the logit model, and positive and negative predictive values indicated that the model has potential in predicting mortality and very poor HRQoL in COPD patients.

## Discussion and conclusion

In this study we used a NBC to detect predictive factors for very poor HRQoL and mortality in COPD patients. HRQoL was measured with a 15D instrument. The outcome variable was dichotomized to poor treatment outcome, poor outcome being expressed as both mortality and very poor HRQoL. We considered this dichotomization justified, because it is clinically important to distinguish patients with with very poor outcome.

The 15D instrument is highly valuated [29]. 15D total scores have been demonstrated to be significantly lower in COPD patients than in age-matched population [30]. The total 15D score has been proved to be significantly correlated with SGRQ (St George's Respiratory Questionnaire), and the breathing subscale of 15D has been significantly correlated with clinical symptoms and functional parameters in COPD patients [31]. 15D scores have been found to change in COPD patients receiving pulmonary rehabilitation, indicating the usefulness of HRQoL in evaluating COPD rehabilitation programs [32].

In the Finnish health care system, the medication payment compensation for COPD is available for patients with FEV permanently less than 40% of the predicted or permanently less than 50% if the have been at least two exacerbation periods or other complications. The payment compensation makes that all patients with those values have passed the same system. It also makes the population constant.

We used a NBC for the analysis. Despite its simplicity, its performance is comparable with conventional [26] or more sophisticated methods [33]. In line with an earlier study [26], NBC’s performance was shown here in comparison to different logit modeling approaches in terms of correspondent or better accuracy and better variable selection. NBCs can manage complicated and limited data, they are easy to use for a clinician, and the results are clearly visualized [26,33-36]. The predictive performance of NBCs can be improved considerably with informative priors [26,37,38]. In these situations, the NBC’s performance may even exceed the performance of logistic regression methods [26,38].

Predicting results may be improved by two ways. Firstly, adding prior information for the model may improve predictions especially in a situation when the model is applied to a new environment. Secondly, finding new predicting variables, for example genetic factors associated with COPD, could be very useful in predicting the outcome. We don't expect that a larger dataset could be very helpful.

Our material consisted of patients derived from the patient flow of pulmonary clinics in two large referral hospitals. We consider that the results can be generalized to COPD patients in secondary care. This study was limited to patients with COPD diagnosis. However, our model is better for predicting health than for predicting morbidity/mortality (very poor HRQoL/death).

Our results cannot be directly compared with previous studies due to the different outcomes. Also, previous studies were performed by using logistic regression methods to identify risk factors. However, co-morbidity, FEV_1_% of predicted, and increased BMI are common predictive factors in our study and in the previous studies. Age over 65 years at onset of the disease protected against death or very poor HRQoL, which was a new finding. This is probably due to milder disease with different disease expansion.

There is a trend towards earlier diagnosis and more active therapeutic interventions in the treatment of COPD to prevent the development of disabling disease [39]. It has become obvious that in addition to lung function measures new tools are needed in the management and monitoring of COPD. The statistical approach used in our study provides a potentially new opportunity to identify patients with poor prognosis.

## Competing interests

One author (OPR) is a member of executive board of Wisane Ltd producing NBC models (http://www.wisane.fi), and one author (EJS) works for and is a shareholder of ESiOR Oy providing, for example, health economic analyses. Other authors declare that there are no competing interests associated with this manuscript.

## Authors’ contributions

TL, AL and MK collected the study material. The analysis was performed by OPR and ES. OPR, ES and TL wrote the manuscript, which was finally accepted by all authors. All authors read and approved the final manuscript.

## Pre-publication history

The pre-publication history for this paper can be accessed here:

http://www.biomedcentral.com/1472-6947/13/34/prepub
